# The influence of genetics on the endocannabinoid system gene expression and relevance for targeting reproductive conditions

**DOI:** 10.1186/s42238-025-00275-x

**Published:** 2025-05-29

**Authors:** Keisuke Tanaka, Akwasi A. Amoako, Sally Mortlock, Peter A. W. Rogers, Sarah J. Holdsworth-Carson, Jacqueline F. Donoghue, Wan Tinn Teh, Grant W. Montgomery, Brett McKinnon

**Affiliations:** 1 Department of Obstetrics and Gynaecology, The Royal Brisbane and Women’s Hospital, Brisbane, QLD Australia; 2 Faculty of Medicine, University of Queensland, Brisbane, QLD Australia; 3 Institute for Molecular Bioscience, University of Queensland, St Lucia, Brisbane, QLD 4072 Australia; 4 Department of Obstetrics and Gynaecology, University of Melbourne, Parkville, VIC 3052 Australia; 5 Gynaecology Research Centre, Royal Women’s Hospital, Parkville, VIC 3052 Australia; 6 Julia Argyrou Endometriosis Centre, Epworth HealthCare, Richmond, VIC 3121 Australia; 7 Department of Biomedical Research, University of Berne, Murtenstrasse 35, CH-3010 Berne, Switzerland

**Keywords:** Endocannabinoids, Gene expression, Expression quantitative trait loci (eQTL), Endometrium, Endometriosis, FABP (fatty acid-binding protein)

## Abstract

**Background:**

Endocannabinoids are small lipid molecules that have critical roles in cellular proliferation and function. They are produced locally with their concentrations controlled via the endocannabinoid system (ECS). The important cellular functions of endocannabinoids have made them and the proteins that modulate their expression targets of potential interest for treatment in many different diseases including gynaecological conditions. There is significant evidence of heredity differences in the response to both exogenous and endogenous cannabinoids that hampers the identification of effective targets. Whether compounds targeting endocannabinoids will be effective therefore may rely on personal differences mediated through genetic architecture. To investigate the source of individual differences, we investigated the effects of genetic variants on the expression of the endocannabinoid system genes at both a systemic and individual tissue level with a particular focus on the female reproductive system and the endometrium.

**Methods:**

We performed this analysis using publicly available datasets, including the 31,684 participants from the eQTLGen database and 838 donors to the GTEx database which includes 49 different sources of tissue, as well as an in-house database of 206 endometrial samples. Analysis of the eQTLGen data identified 22,020 eQTLs that influenced 43 of the selected 70 ECS genes.

**Results:**

A comparison across 49 different tissues that included at least 70 different individuals in the GTEx dataset identified eQTL for 69 of the 70 different genes, confirming a tissue-specific influence. Comparisons among 11 different physiological system indicated that the female reproductive system was associated with a fewer number of eQTLs. Finally, in the endometrium, we detected Bonferroni significant genetic effects on one individual gene fatty acid binding protein 3 (*FABP3*), an intracellular transporter that delivers endocannabinoids to the enzyme responsible for its inactivation, with a further 14 independent FDR significant eQTL for 13 ECS genes.

**Conclusions:**

This is the first study to investigate the effects of genetic variants on the ECS gene transcription and indicates genetic variants have significant influence that are unique to each tissue. Our results highlight the effect of individual variation and the impact endocannabinoid based therapies may have on different tissue and physiological systems.

**Supplementary Information:**

The online version contains supplementary material available at 10.1186/s42238-025-00275-x.

## Background

Endocannabinoids are endogenous lipid-based molecules that influence cellular function. The most well-characterised endocannabinoids are anandamide (AEA) and 2-arachdonoylglycerol (2-AG). Their expression is mediated by a complex array of proteins and enzymes that together make up the endocannabinoid system (ECS) (Lu and Mackie 2016). The ECS is one of the most crucial systems in the human body, engaged in numerous physiological processes including nociception, mood regulation, cognitive function, neurogenesis, appetite, and lipid metabolism. It controls cellular functions including cell migration (Gentilini et al. 2010; McHugh et al. 2012), cell proliferation (Leconte et al. 2010), cell survival (Bilgic et al. 2017) and inflammation (Resuehr et al. 2012; Iuvone et al. 2008). With such a wide array of functions, the ECS system has received increasing attention as a promising therapeutic target for many medical conditions including gynaecological pathologies such as endometriosis, infertility, ectopic pregnancy and miscarriage. Ultimately however, the development of novel drugs has turned out to be a challenging task, potentially due to the complex nature of the endocannabinoid system, crosstalk with other systems, and possibly the influence of genetic variants on endocannabinoid activity.

Cannabis sativa, which contains the phyto-cannabinoid D9 tetrahydrocannabinol (THC) has long been used for medical purposes, particularly for the control of pain (Almogi-Hazan and Or 2020), although the efficacy of cannabis in clinical trials for chronic pain varies (Aviram and Samuelly-Leichtag 2017; Stockings et al. 2018). A meta-analysis reported that genetic influences accounted for 48% of the total variance for cannabis use initiation and 51% for problematic use (Verweij et al. 2010). Twin-based studies have confirmed the role of genetics in cannabis addiction (Revised American Society for Reproductive Medicine classification of endometriosis: 1996 1997) and evidence from genome-wide association studies (GWAS) has identified genetic regions that mediate this addiction risk (Hillmer et al. 2021). A thorough understanding of the genetic regulation of endocannabinoid activity is lacking. Whether a similar genetic influence on other endocannabinoids occurs has not yet been assessed, but could be achieved through altered regulation of ECS genes. An influence of genetic variants on genome-wide gene expression at regions termed expression quantitative trait loci (eQTL) is associated with 15–100% of the variance in gene expression (Powell et al. 2012), which underlies many human traits and diseases (Control and C. Genome-wide association study of 14, 000 cases of seven common diseases and 3, 000 shared controls. 2007; Gamazon et al. 2018). eQTLs could contribute to the regulation of the ECS at both a system and local level.

Endometriosis is a gynaecological condition defined by the presence of endometrial-like tissue outside the uterus and it is accompanied by a chronic inflammatory reaction. It is associated with symptoms such as dysmenorrhoea, pelvic pain, dyspareunia, dyschezia and infertility (Giudice and Kao 2004; Moradi et al. 2014), and is the most common cause of chronic pelvic pain in women (Fauconnier and Chapron 2005). Endometriosis affects 6 to 10% of women of reproductive age, 50 to 60% of women and teenage girls with pelvic pain, and up to 50% of women with infertility (Goldstein et al. 1980; Eskenazi and Warner 1997). It significantly impacts the social participation and mental health of those affected (Nnoaham et al. 2011). The ECS has been identified as a potential therapeutic target for endometriosis (Tanaka et al. 2020) and the use of cannabis has been shown to be effective in managing pain for some, but not all women (Hillmer et al. 2021). We have recently charted the significant variation in ECS genes in the endometrium across the different stages of the menstrual cycle and identified significant levels of individual gene expression variation.

Although genetic influences on the activity of endogenous and exogenous endocannabinoids are reported, the effect of genetic variants on the gene expression of the ECS has not previously been systematically examined. The objective of this study was to analyse the influence of genetic variants on genes associated with the ECS at both a systematic level in the bloodstream and in individual tissue types including the endometrium to determine the influence on gene expression and identify potential novel targets that should be considered for both targeting and efficacy in individual patients. Given the well-documented variation in endocannabinoid activity, understanding the impact of genetics will be of relevance in determining the suitability of patients to endocannabinoid-based treatment modalities.

## Methods

### Selection of the ECS genes

A priori gene selection was performed through a literature search using the search term ‘endocannabinoid’, and combinations of ‘pathway’, ‘synthesis’, ‘metabolism’ or ‘transport’. These genes were published previously (Tanaka et al. 2022) and can be found in Supplementary Table 1. All genes relevant to the control of the ECS were catalogued and the ENSEMBL gene transcript IDs were determined, and gene expression data was extracted from the curated databases. ECS genes of interest were split into various categories based on their main function in the ECS including synthesizing enzymes, receptors, transporters, and degradation enzymes. In total 70 potential genes known to play a role in the ECS were assessed.

### eQTLGen analysis of endocannabinoid genes

To determine the genetic effects on gene expression at both systemic and local tissue level we extracted summary-level statistics data from the eQTLGen (Võsa et al. 2021), that includes gene expression and genotyping data from 31,684 participants through blood or peripheral blood mononuclear cells (PBMC) from 37 different patient cohorts. We restricted our analysis to the 70 a priori selected ECS genes.

### eQTL analysis in local tissue using the GTEx dataset

To determine the presence of shared eQTLs across tissue within the ECS we extracted summary-level statistics from the GTEx version 8 data that includes genotype and gene expression data from 838 donors and 17,382 samples from 52 tissues and two cell lines (Battle et al. 2017). GTEx is a consortium focused on identifying the influence of genetics on gene expression in a complex array of individual tissues. We analysed 49 tissues or cell lines that had at least 70 individuals and performed the analysis on the 70 a priori selected genes.

### Endometrial sample eQTL analysis

We analysed RNA-seq and genotype data from a previously published study (Mortlock et al. 2020). Inclusion criteria were European ancestry and within reproductive age (18–49) and exclusion criteria were the use of hormonal medication within 3 months prior to surgery, abnormalities in histopathological examination, ambiguous disease status or cycle stage. Menstrual cycle dating was performed via a histological assessment of each endometrial biopsy according to the Noyes criteria (Noyes et al. 1960). DNA extraction, genotyping, and imputation, as well as RNA-seq and read alignment was performed as described previously (Battle et al. 2017).

RNA-seq libraries had a mean depth of 37,490,673 for 178 samples and 120 bp paired-end reads on an Illumina Hi Seq 2000 (Illumina, USA) for a mean depth of 40,818,062 reads for 28 samples. eQTL analysis was performed by sub-setting the 70 a priori selected endocannabinoid genes and assessing the association with genetic variants. Analysis was restricted to genes expressed in greater than > 90% of samples and individual-level genotype data for 6,230,993 SNP were included in the analysis.

### Statistical analysis

Statistical analysis of all data including the eQTLGen, GTex and endometrial data was performed with R and significance was assessed at both the false discovery rate (FDR) and Bonferroni’s cut-off applying a genome-wide threshold. *cis*-eQTLs were defined as SNPs located within ± 250 kb from gene start and stop position, and a *cis*-eQTL analysis was performed with a linear regression model using the MatrixeQTL R package v2.2 (Shabalin 2012).

## Results

### Systemic genetic influence on the ECS

We extracted summary statistics from the eQTLGen database, a consortium focused on investigating the genetic influence of gene expression in peripheral blood. We restricted our analysis to the a priori set of 70 genes selected from the literature and responsible for controlling endocannabinoid concentrations (Supplementary Table 1) and performed a *cis-*eQTL analysis. We identified 33,183 eQTLs that passed FDR significance influencing 49 of the 70 selected genes. In an assessment of significance using Bonferroni’s cut-off we identified 22,020 eQTLs that influence 43 genes, identifying a genetic influence on approximately 60–70% of the ECS genes in blood.

**Fig. 1:**
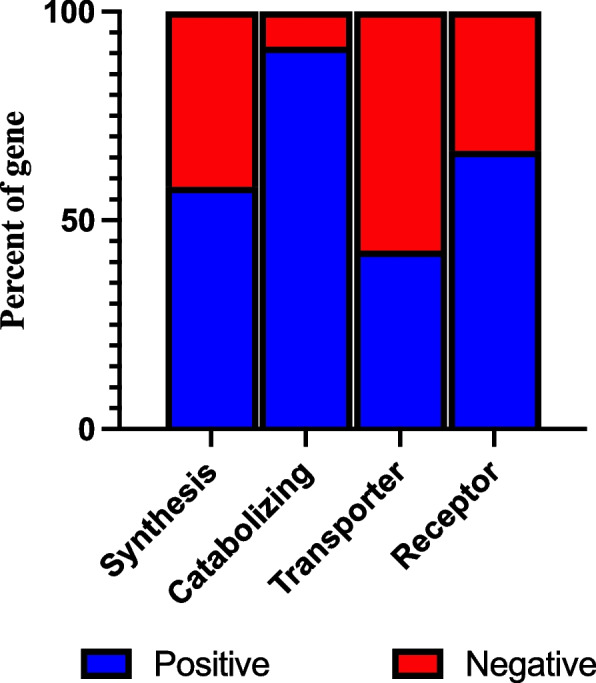
ECS genes effected by eQTLs in eQTLGen. We assessed the eQTLs that effected the 70 ECS genes and separated them based on their function within the system. The analysis identified very high proportion of the Catabolizing enzymes to be significantly impacted by genetic background

We have previously analyzed the expression of these genes by separating them into 4 distinct groups based on their role within the endocannabinoid system (Tanaka et al. 2022). Using the FDR cut off we identified 18,794 eQTL influencing 31 out of the 45 synthesizing enzymes, 12,278 eQTLs influencing 11 out of the 12 catabolizing enzymes, 453 eQTL influencings 3 out of 7 transporter proteins and 1,658 eQTL influencing 4 out of 6 receptor genes **(**Table 1). Of these groupings, there appeared to be a strong genetic regulation on the catabolizing enzymes group compared to other functions, with eQTL for over 90% of these genes (Fig. 1).

**Table 1:** A list of 49 ECS genes associated with FDR significant eQTLs in eQTLGen

Synthesizing			Catabolizing	Transporter	Receptor
Isoforms of PLA2	Isoforms of PLC	Others			
PLA2G12A	PLCB1	ABHD4	ABHD12	FABP3	CNR1
PLA2G2C	PLCB2	DAGLA	ABHD6	FABP5	CNR2
PLA2G2D	PLCB3	GDE1	ALOX12	FABP6	GPR55
PLA2G4A	PLCD1	GDPD1 (GDE4)	ALOX15		TRPV1
PLA2G4B	PLCD3	GDPD3 (GDE7)	ALOX5		
PLA2G4C	PLCD4	NAPEPLD	CYP2D6		
PLA2G6	PLCE1	NAT1	CYP4F2		
PLA2G7	PLCG1	PLD1	FAAH		
	PLCG2	PLD2	MGLL		
	PLCH1	PTPN22	NAAA		
	PLCH2		PTGS2 (COX-2)		
	PLCL1				
	PLCL2

### Tissue-specific genetic regulation of the ECS

Endocannabinoids are short-lived molecules that are produced locally at the cell surface membrane and can influence individual tissues differently. In addition to determining whether underlying genetic architecture can influence endocannabinoid concentrations, we also assessed tissue-specific regulation by analyzing data from the GTEx database. Across all the tissue examined, *PLA2G2E* was the only gene for which no eQTL was observed in any of the 49 tissues or cell lines. A list of genes that were under significant influence of eQTL in each tissue is presented in Supplementary Table 2.

**Fig. 2:**
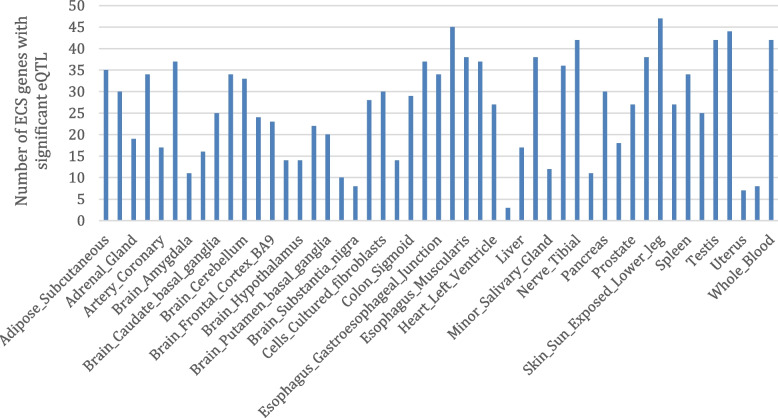
ECS genes associated with significant eQTL in different tissues. Analysis of the GTex database containing genotype and gene expression values for 47 different tissues identified variation in the number of eQTL-regulating ECS-associated genes

**Fig. 3:**
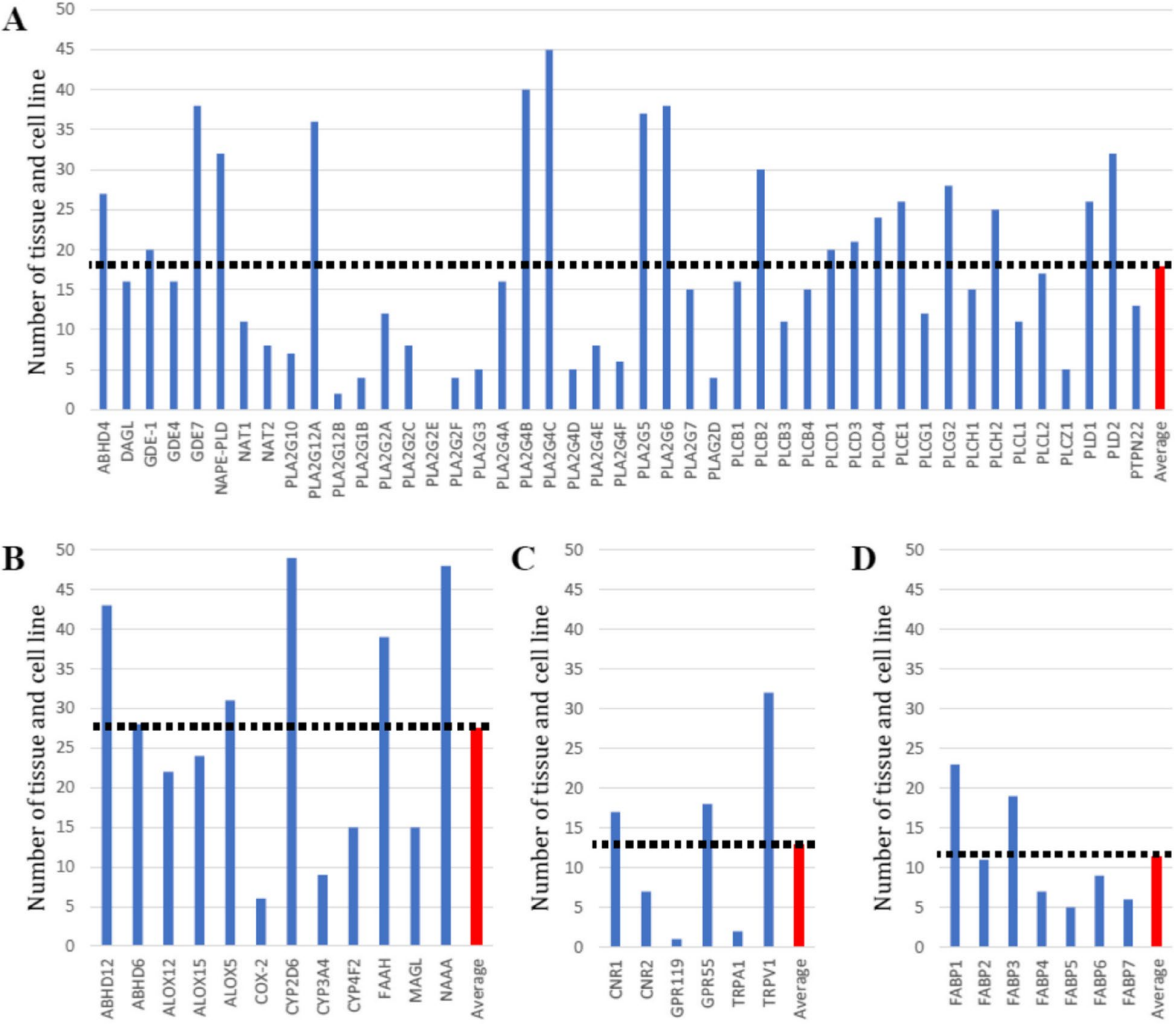
Tissue and cell line in which the ECS genes were associated with significant eQTL in GTEx database. Analysis of individual genes in different tissues indicated the genetic regulation of some genes was more common than others. This analysis was split by genes that encode **A**) Synthesising genes, **B**) Catabolising genes, **C**) Receptor genes and **D**) Transporter genes. The read bar and dotted line indicate the Mean number of eQTLs for each tissue

This analysis found eQTLs were highly replicable across tissues. Sun-exposed skin (47), esophageal mucosa (45), thyroid (44), whole blood (42), testis (42) and tibial nerve (42) were associated with the largest number of ECS genes that were under significant genetic regulation (Fig. 2). In contrast, the kidney cortex (3), uterus (7), substantia nigra (8), vagina (8) and spinal cord (10) were associated with the least number of ECS genes with significant eQTL. Separated by biological function, 45 synthesizing enzymes had significant eQTLs in 17.9 tissues on average (Fig. 3A), 12 catabolizing enzymes had significant eQTLs in 27.4 tissues (Fig. 3B), 6 receptors had significant eQTLs in 12.8 tissues (Fig. 3C), and 7 transporters had significant eQTLs in 11.4 tissues (Fig. 3D). Genetic regulation of catabolizing enzymes was observed in the most tissues, with eQTLs identified across all 49 of the different tissues examined.

**Fig. 4:**
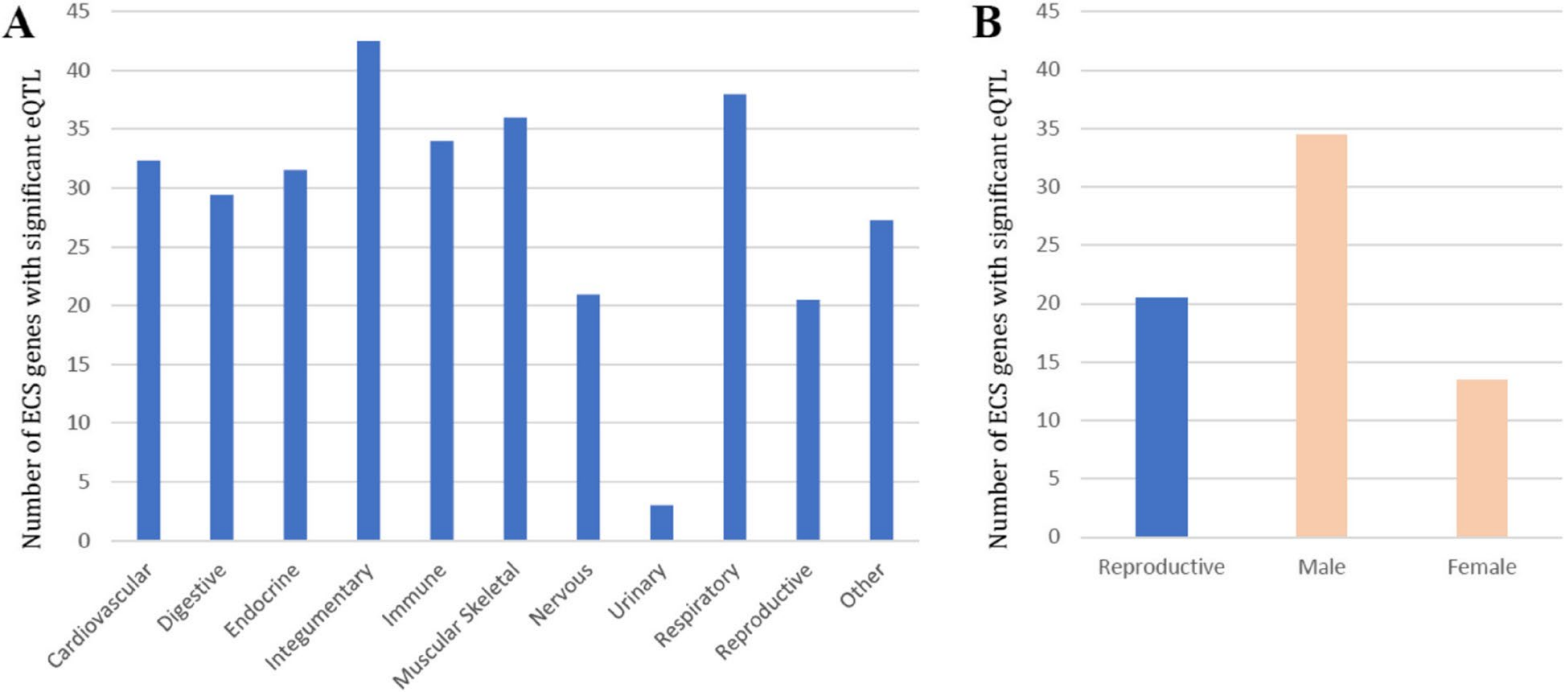
The number of ECS genes associated with significant eQTL separated by physiological system **A**) in 11 physiological systems. **B**) The reproductive system is divided into male and female reproductive systems

We subsequently divided the 49 tissues and cell lines into 11 categories based on physiological systems; cardiovascular, digestive, endocrine, integumentary, immune, musculoskeletal, nervous, urinary, respiratory, reproductive and other. Integumentary systems (consisting of sun-exposed and non-sun-exposed skin) involved the largest number of ECS genes that were influenced by genotypes (mean 42.5 genes), followed by the respiratory system (consisting of lung, mean 38 genes) and musculoskeletal (consisting of muscle skeletal, mean 36 genes) (Fig. 4). The reproductive system (consisting of breast mammary tissue, ovary, uterus, vagina, prostate and testis) showed the second smallest number of ECS genes that were associated with significant eQTLs (mean 20.5 genes), after the urinary system which consisted only of kidney cortex (mean 3). Furthermore, the female reproductive system showed a smaller number of ECS genes associated with eQTLs (mean 13.5) compared to the male reproductive system (mean 34.5 genes).

### Genetic regulation of ECS in the endometrium

We subsequently examined eQTL in our in-house dataset of endometrial samples (a tissue not present in the GTEx database). Of the 70 ECS genes selected a priori for examination, there was 1 gene with no discernible expression in any samples (*PLA2G2E*), 61 were expressed in 3 or more samples, and 40 were expressed in greater than 90% of all samples (Supplementary Table 3). We focused the subsequent analysis on these 40 genes consistently expressed. Of these 40 genes included in the analysis, 29 genes encoded synthesizing enzymes, 8 encoded catabolizing enzymes, 2 encoded transport proteins and 1 encoded endocannabinoid membrane receptor. Bonferroni correction for multiple testing detected genetic effects on gene expression in endometrium for one independent *cis-*eQTL, an endocannabinoid transport protein, fatty acid-binding protein 3 (*FABP3*) gene; rs115552871 on chromosome 1 (p = 4.85 × 10^–10^) (Table 3).

**Fig. 5:**
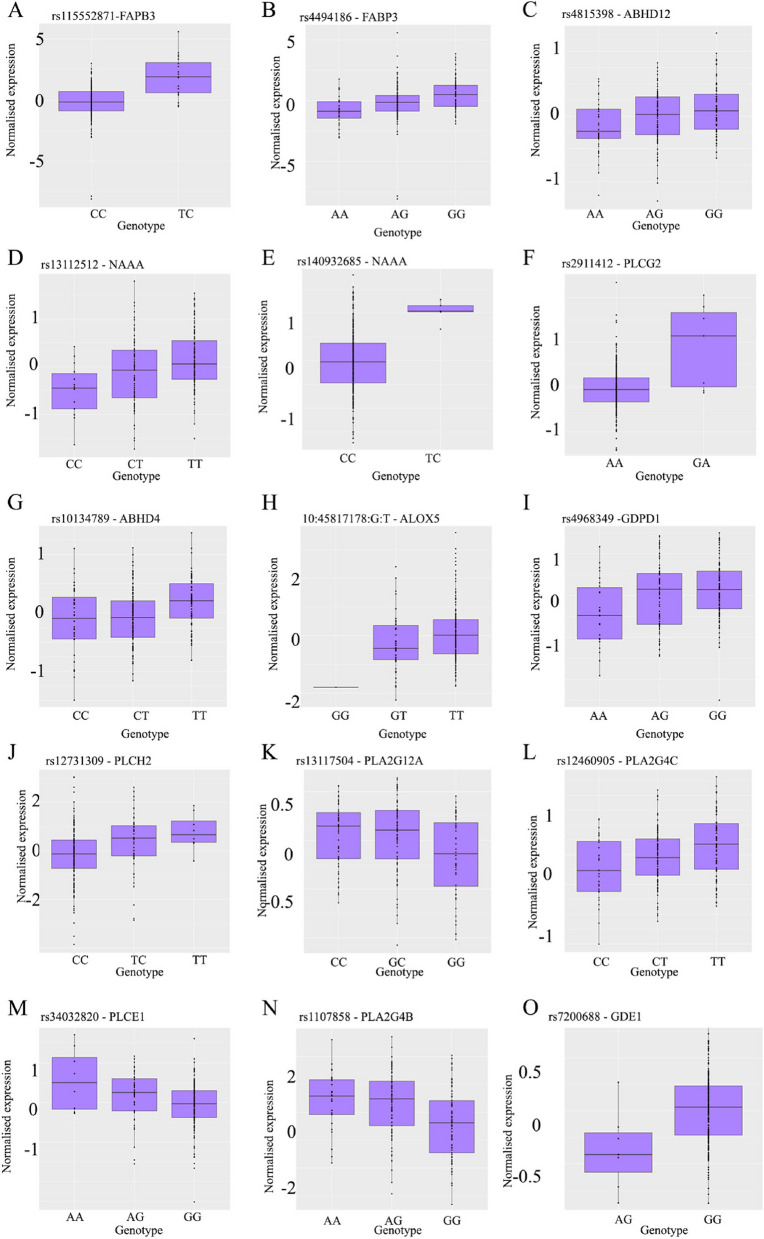
Genetic effects on normalized gene expression for 2 Bonferroni and 13 FDR significant eQTLs identified in the endometrial tissue. A genome-wide Bonferroni significant eQTL was identified for **A**) rs115552871- FAPB3, with subsequent FDR significant eQTLs observed for **B**) rs4494186- FAPB3, **C**) rs4815398-ABHD12, **D**) rs13112512-NAAA, **E**) rs140932685, F) rs2911412-PLCG2, **G**) rs10134789, **H**) 10:45817178G:T -ALOX5, **I**) rs4968349, **J**) rs12731309 **K**) rs13117504 = PLA2G12A, **L**) rs12460905, **M**) rs34032820, **N**) rs1107858, rs7200688-GDE1

There were an additional 14 independent FDR significant *cis*-eQTLs (p < 5.32 × 10^–4^) for 13 ECS genes (Table 2). Nine genes encode enzymes responsible for endocannabinoid production (3 isoforms of PLC including *PLCH2*, *PLCG2* and *PLCE1*, 3 isoforms of PLA2 including *PLA2G12A*, *PLA2G4B* and *PLA2G4C*, two GDEs 1 and 4 [also known as GDPD1], and *ABHD4*), 3 genes encode catabolizing enzymes (*NAAA*, *ABHD12* and *ALOX5*), and a transporter *FABP3* gene. None of the endocannabinoid receptor genes had significant eQTLs in the endometrium. Two secondary signals were identified for *FABP3* and *NAAA*. Normalized gene expression at each genotype for these 13 primary and 2 secondary signals for each genotype are presented in Fig. 5. Finally a list of ECS genes with significant eQTL in the ovary, uterus and ovary from the GTEx and in the in-house endometrial database is shown in Table 3.

**Table 2:** 15 significant eQTL for 13 ECS genes in the endometrium

SNP	Chr	BP	A1	A2	Freq	Probe	Probe_Chr	Probe_bp	Gene	b	se	p	FDR_Sig	Bonf_Sig	Signal	Conditional_P
rs115552871	1	31,330,095	C	T	0.95708	ENSG00000121769	1	31,365,625	FABP3	-2.2049404	0.33529638	4.85E-10	Yes	Yes	Primary	NA
rs13112512	4	75,946,755	T	C	0.7446	ENSG00000138744	4	75,913,657	NAAA	0.28478151	0.06108454	6.00E-06	Yes	No	Primary	NA
rs4815398	20	25,256,702	G	A	0.5558	ENSG00000100997	20	25,294,743	ABHD12	0.17288131	0.03788992	9.21E-06	Yes	No	Primary	NA
rs12731309	1	2,420,707	C	T	0.8326	ENSG00000149527	1	2,425,980	PLCH2	-0.6017947	0.13449833	1.34E-05	Yes	No	Primary	NA
rs13117504	4	109,737,700	C	G	0.5536	ENSG00000123739	4	109,709,989	PLA2G12A	0.09834292	0.02241623	1.93E-05	Yes	No	Primary	NA
rs7200688	16	19,455,737	G	A	0.98283	ENSG00000006007	16	19,501,689	GDE1	0.42662428	0.09775976	2.13E-05	Yes	No	Primary	NA
rs1107858	15	41,676,028	G	A	0.6438	ENSG00000243708	15	41,837,775	PLA2G4B	-0.5557567	0.12836048	2.45E-05	Yes	No	Primary	NA
rs2911412	16	82,197,974	A	G	0.98283	ENSG00000197943	16	81,739,097	PLCG2	-0.8189833	0.19225365	3.26E-05	Yes	No	Primary	NA
rs12460905	19	48,185,426	T	C	0.5923	ENSG00000105499	19	48,047,843	PLA2G4C	0.21856361	0.05302346	5.68E-05	Yes	No	Primary	NA
rs34032820	10	94,368,119	G	A	0.8455	ENSG00000138193	10	94,030,812	PLCE1	-0.2712103	0.06936836	0.00012982	Yes	No	Primary	NA
rs10134789	14	22,592,920	T	C	0.5708	ENSG00000100439	14	22,598,237	ABHD4	0.17349972	0.04493194	0.000156	Yes	No	Primary	NA
10:45,817,178:G:T	10	45,321,730	T	G	0.90129	ENSG00000012779	10	45,374,176	ALOX5	0.51439736	0.13778999	0.0002519	Yes	No	Primary	NA
rs4968349	17	59,326,591	G	A	0.6545	ENSG00000153982	17	59,220,467	GDPD1	0.22601095	0.06210172	0.00035497	Yes	No	Primary	NA
rs4494186	1	31,385,000	G	A	0.6116	ENSG00000121769	1	31,365,625	FABP3	0.78785121	0.1629067	2.79E-06	Yes	No	Secondary	3.00E-04
rs140932685	4	75,828,201	C	T	0.98712	ENSG00000138744	4	75,913,657	NAAA	-0.83737	0.25093278	1.02E-03	No	No	Secondary	3.79E-04

**Table 3:** A list of the ECS genes with significant eQTL across the female reproductive system

GTEx			In-house database
**Ovary**	**Uterus**	**Vagina**	**Endometrium**
**ABHD12**	**ABHD12**	**CYP2D6**	**ABHD4**
**ALOX12**	**ALOX5**	**CYP4F2**	**ABHD12**
**CYP2D6**	**CYP2D6**	**NAAA**	**ALOX5**
**NAAA**	**NAAA**	**NAPE-PLD**	**FABP3**
**PLA2G2A**	**NAT2**	**NAT1**	**GDE1**
**PLA2G4B**	**PLA2G4C**	**PLA2G12A**	**GDPD1**
**PLA2G4C**	**PLCB2**	**PLA2G4C**	**NAAA**
**PLA2G5**		**TRPV1**	**PLA2G12A**
**PLA2G6**			**PLA2G4B**
**PLCE1**			**PLA2G4C**
**TRPV1**			**PLCE1**
			**PLCG2**
			**PLCH2**

For those 13 ECS genes for which there were independent FDR significant *cis*-eQTLs (p < 5.32 × 10^–4^), *NAAA* was the most commonly influenced by genotypes across 49 tissues and cell lines (48 out of 49 tissues and cell lines). Other commonly influenced ECS genes were *ABHD12* (43 tissues), *ALOX5* (31 tissues), *PLA2G4C* (45 tissues), and *PLA2G4B* (40 tissues). In contrast, *GDE4* was the least likely gene to be influenced by genetic variants (16 tissues), followed by *FABP3* (19 tissues).

## Discussion

Our analysis of genetic effects on genes within the ECS across tissue using the eQTLGen and GTEx database indicated variation in both the extent and identity of genes influenced in each tissue type. The in-house endometrial dataset analysis determined that 49 out of 70 ECS genes were significantly impacted by genetic variants indicating a potential influence on endocannabinoid bioactivity within the endometrium through an impact on intracellular transport and non-canonical degradation pathways. Together these data indicate a role for genetic regulation on endocannabinoid activity with the potential to control local concentrations. Genetic influence should be considered when assessing novel compounds and their efficacy.

Comprehensive analysis of the eQTLGen database has now shown that there are > 16,000 genes influenced by common genetic variants, representing approximately 80% of genes expressed in most tissue (Võsa et al. 2021), highlighting the important influence of genetics on gene expression across the entire genome. In this study we found only 61.4% of the ECS genes were significantly impacted at the systemic level, suggesting a slightly lower level of influence compared to what could be expected, based solely on the number of genes influenced. When separating genes based on their function it appears the major influence occurred on enzymes that were responsible for breaking down endocannabinoids with 11 out 12 impacted (91.7%), including *FAAH, MGLL, NAAA* and *COX-2,* which may ultimately influence the bioactive time of endocannabinoids in the blood and have implications for the pharmacokinetic effects of both endogenous and exogenous cannabinoids. Previous evidence has indicated there are differences in time-dependent clearance of THC between individuals (Huestis 2007).

From the analysis of these genes in the GTEx database, we were able to assess the genetic effects on gene expression of 838 individuals in a total of 52 different tissues. This analysis indicated that many of the ECS genes were influenced by genetic variation across different tissues. There was a variation in both the number and identity of genes that were influenced in each tissue, suggesting local differences in the impact on endocannabinoids concentrations. The physiological system with potential for the most significant regulation by genetic effects was the skin while the female reproductive system showed a smaller number of ECS genes with significant eQTLs. However, a variation in the sample size for the different tissues in the GTEx database should be noted. The Kidney which was associated with the least number of ECS genes with significant eQTL and the female reproductive organs had smaller sample sizes. Comparison across the female reproductive system organs and the endometrium identified that NAAA was under the genetic regulatory influence across all organs.

Previous studies have also confirmed clinical significance for genetic variants that influence the ECS. In candidate gene studies, SNPs in *CNR1* (rs2023239) and *FAAH* (rs324420) have been associated with substance abuse and functional changes in cannabinoid regulation. This mutation in FAAH produces a non-synonymous 385C variant and a mutant FAAH enzyme characterised by reduced cellular stability (Onaivi et al. 2002). Gene-based tests identified four genes associated with lifetime cannabis use, *NCAM1, CADM2, SCOC and KCNT2* (Stringer et al. 2016), although their role in the ECS system is unclear. In a genome-wide association study (GWAS) that included 32,330 patients from 13 cohorts, and a replication set of 4 cohorts, with 5,627 patients found no genes reached genome-wide significance. A more recent GWAS including 20,916 case samples with 363,116 control samples and focussing on cannabis use disorder identified two genome-wide significant loci rs7783012 near FOXP2 and rs4732724 near CHRNA2 and EPHX2 (Johnson et al. 2020).

Given the potential interest in endocannabinoid treatments for reproductive conditions that cause pelvic pain, such as endometriosis, we assessed the genetic effect on genes in the endometrium. The ECS are expressed in abundance in the female reproductive tract and are implicated in both pain and inflammation (Guindon and Hohmann 2009). We identified one eQTL that passed Bonferroni significance; *FABP3* rs115552871 on chromosome 1. FABPs are intracellular carrier proteins that deliver endocannabinoid AEA from the plasma membrane to intracellular FAAH for inactivation (Kaczocha et al. 2009). Although AEA uptake and subsequent hydrolysis were elevated with FABP5 and FABP7 and not with FABP3 using cell lines derived from monkey kidney cells (COS-7) and mouse nerve cells (N18TG2) (Kaczocha et al. 2009), AEA and 2-AG were found to have higher affinities to human FABP3 compared to FABP5 and FABP7 (Elmes et al. 2015). These genotypes which significantly affect *FABP3* gene expression may lead to critical differences in AEA and further contribute to a genetic influence on bioactive timeline for endocannabinoids.

Our findings of the effects of individual genotypes on the expression of the ECS may suggest possible clinical implications with the use of exogenous cannabinoids. Cannabis has a long history of use for various conditions including dysmenorrhea, a cardinal symptom of endometriosis. Research has demonstrated that exogeneous cannabinoids such as THC (Aggarwal 2013; Rahn and Hohmann 2009), ∆9-Tetrahydrocannabivarin (THCV) (Bolognini et al. 2010) and Cannabidiol (CBD) exert analgesic and anti-inflammatory effects (Bouaziz et al. 2017), and cannabis scored the highest self-rated effectiveness among a list of self-management modalities by women with endometriosis (Armour et al. 2019). Resembling endocannabinoids, THC and CBD were also shown to bind to human FABPs including FABP3 (Elmes et al. 2015). Identifying genetic variants that influence FABP3 expression and subsequent endocannabinoid bioactivity may have a future role in formulating personalized treatment, and predicting treatment response and side effects for those who are prescribed exogenous cannabinoids.

In addition to the transporter protein FABP3, we found that gene expression of a number of synthesizing and catabolizing enzymes in the endometrium are also under the influence of eQTLs. There are four routes for AEA synthesis. The most widely accepted pathway is the single-step, direct synthesis from hydrolysis of its membrane precursor NAPE by the enzyme NAPEPLD (Marzo et al. 1994; Wang et al. 2006; Ueda et al. 2005; Okamoto et al. 2004). The remaining three pathways are two-step processes. Firstly, conversion of NAPE to phospho-anadamide (p-AEA) by phospholipase C (PLC) followed by dephosphorylation by the protein tyrosine phosphatase N22 (PTPN22) (Liu et al. 2006, 2008). Secondly, hydrolysis of NAPE to 2-lyso-NAPE by phospholipase A2 (PLA2) and then to AEA by GDE4 or 2-lyso-phospholipase D (LysoPLD) (Sun et al. 2004; Tsuboi et al. 2013). Finally, conversion of NAPE to glycerophospho-arachidonyl ethanolamide (Gp-AEA) by double-deacylation by the enzyme α/β hydrolase 4 (ABHD4) followed by a specific phosphodiesterase (glycerophosphodiester phosphodiesterase 1: GDE1) (Simon and Cravatt 2006, 2010). Our analysis indicates that gene expression for the majority of AEA synthesizing enzymes that are involved in the two-step degradation processes are under genetic regulation (3 isoforms of PLC, 3 isoforms of PLA2, two GDEs 1 and 4, and ABHD4).

Endocannabinoid signaling is controlled through a balance of on demand synthesis and prompt catabolism (Basavarajappa 2007; Howlett et al. 2011), and endocannabinoids continue to exert their activity until degraded. Fatty acid amide hydrolase (FAAH) is the main enzyme responsible for degradation of AEA (Cravatt et al. 1996; Giang and Cravatt 1997). In addition, AEA is subjected to oxygenation by a number of enzymes including cyclooxygenase-2 (COX-2) (Rouzer and Marnett 2011; Kozak et al. 2002), 5-, 12- and 15-lipoxygenase (5-/12-/15-LOX) (Hampson et al. 1995; Stelt et al. 2000), and several cytochrome P450 monooxygenases (P450s) (Snider et al. 2010; Urquhart et al. 2015). Monoacylglycerol lipase (MAGL) is the chief 2-AG degrading enzyme (Marzo et al. 1999a, 1999b), and there are at least three other hydrolases: FAAH, α/β-hydrolase domain containing (ABHD) 6 and ABHD12 (Blankman et al. 2007). Our results revealed that in the endometrium while there is no genetic influence on the expression of the main degrading enzymes, FAAH and MAGL, eQTLs alter the expression of 3 catabolizing enzymes, NAAA, ABHD12, and ALOX5. NAAA deactivates palmitoylethanolamide (PEA) which is involved in the control of pain and inflammation, and pharmacological inhibition of NAAA has been reported for parkinsonism and neuropathy (Palese et al. 2022; Toma et al. 2021).

One of the limitations of the study is the homogeneity of the study population. While the eQTLGen consortium incorporates 37 datasets, many are based on individuals living in urban centres primarily from Europe and Asia. More than 85% of the GTEx version 8 and all individuals in our database of endometrial samples are of European ancestry. Consequently, most eQTL studies have been based on individuals of European ancestry and are underrepresented in other populations. As eQTL mapping results can vary substantially across populations with diverse genetic backgrounds due to differences in allele frequencies and linkage disequilibrium patterns (Kelly et al. 2017), establishment of database that represent diverse population is warranted.

In this study, we have assessed the gene expression that encodes proteins that regulate the ECS system and have identified, multiple and significant influences on gene expression that could impact endocannabinoid bioactivity and consequences. We have investigated the largest available public datasets and identified significant genetic influences. We have not directly assessed endocannabinoid expression in the local tissue. Instead, we have focused on the genes that regulate the system as a proxy for endocannabinoid concentrations. This is because firstly endocannabinoids are lipid molecules with short-lived duration and assessing their concentration at one particular time point does not account for the significant variation that can occur in a time-dependent manner, making the influence of genetic effects difficult to establish. Secondly, we are interested in understanding the important molecules that regulate the endocannabinoid regulation and that may provide potential drug targets.

Human genetic diversity are major determinants of interindividual variability in susceptibility to diseases, response to therapy, and the clinical outcomes (Marian 2020). We investigated the effects of genetic variants on the expression of the ECS genes at both a systemic and individual tissue level. The analysis of the GTEx database indicated that while many ECS genes were influenced by genetic variation across different tissues, the female reproductive system was associated with one of the least degrees of influence from genetic variants, suggesting that it is resistant to interindividual variability. The most significant eQTL in the endometrium involved *FABP3,* intracellular carrier proteins that deliver endocannabinoid AEA from the plasma membrane to intracellular FAAH for inactivation, identifying it as the potential candidate as a predictor of susceptibility to endometrial diseases or response to endocannabinoid system based therapeutics in such diseases.

## Conclusions

In summary, this is the first study to investigate the effects of genetic variants on the regulation of genes that encode and regulate the ECS. We have assessed an exhaustive list of genes that have been implicated in the ECS and have investigated large-scale publicly available data, as well as our existing endometrial data to determine the effects of genetic regulation in the ECS system across different tissue types including tissues for diseases that have been suggested to benefit from ECS modulation. Together with the existing knowledge of the complex ECS and their interaction with exogenous cannabinoids, this study provides pioneering information on potential clinical implications of genetic background that may have significant local and systemic effects on endocannabinoid modulation.

## Supplementary Information

Supplementary Material 1.

Supplementary Material 2.

Supplementary Material 3.

## Data Availability

Not applicable.

## Declarations

### Completing interests

The authors declare that they have no completing interests.
